# Allele and genotype frequencies of variants in P450 cytochromes, transports, and DNA repair enzymes in the Dominican Republic population

**DOI:** 10.3389/fphar.2024.1494482

**Published:** 2025-03-05

**Authors:** Elizabeth Pérez-Duval, Berniza Calderón, Marlen Izquierdo, José A. Herrera-Isidrón, Elizabeth Reyes-Reyes, Alejandro Herrera, Manuel Soto, Alba Beltré, Idania Rodeiro-Guerra

**Affiliations:** ^1^ School of Medicine, Instituto Tecnológico de Santo Domingo (INTEC), Santo Domingo, Dominican Republic; ^2^ Research Unit, Centro Médico de Diabetes, Obesidad y Especialidades (CEMDOE), Santo Domingo, Dominican Republic; ^3^ Research Committee, Sociedad Dominicana de Endocrinología y Nutrición (SODENN), Santo Domingo, Dominican Republic; ^4^ Institute of Material Science and Technology (IMRE), University of Havana, Havana, Cuba; ^5^ Department of Pharmacology, Institute of Marine Sciences (ICIMAR), Havana, Cuba; ^6^ Cuban National Center of Biodiversity, Institute of Ecology and Sistematic of Cuba, Havana, Cuba

**Keywords:** genetic variants, SNVs, pharmacogenetic, Dominic Republic population, admixed population

## Abstract

**Introduction:**

Single-nucleotide variants (SNVs) give rise to important inter-individual and inter-ethnic variabilities in the metabolism and disposition of several therapeutic agents and may cause differences in the treatment response to clinically important drugs like antiarrhythmics, antidepressants, antihistamines, and antipsychotics, among others. Information about the prevalence of variants in the Dominican Republic population is still limited. The aim of this study was to describe the frequency distribution of 32 SNVs from 14 genes with pharmacogenetic interest within a sample of 150 unrelated healthy individuals.

**Methods:**

Genotype and allele frequencies were determined, and pairwise Wright’s F_ST_ statistic was evaluated.

**Results:**

Hardy–Weinberg equilibrium deviations were found in seven loci from *CYP2D6* (rs16947, rs3892097, rs1058164, rs1135840, and rs28371725) and *CYP2C19* (rs12769205 and rs4244285) genes. The minor allele frequencies ranged from 0.01 to 0.50 values in the xenobiotic biotransformation enzymes and transporter genes. The average admixture estimates were 51.6%, 39.5%, and 8.9% for European, African, and Amerindian ancestries, respectively. Pairwise F_ST_ analysis revealed that Dominicans displayed genetic similarity to Latin American populations, especially those with Afro-Caribbean ancestry, given the selected variants. Higher differences were identified from East and South Asians, Europeans, and Africans, in which several values above the F_ST_ threshold for moderate differentiation were identified within variants in *CYP2C, CYP3A, CYP1A1, ABCB1, SLC45A2, XRCC1*, and *XRCC3* genes.

**Conclusions:**

These results should allow establishing the clinical relevance of pharmacogenetic testing in variant alleles related to drug transport and metabolism genes in this population.

## 1 Introduction

The study of genetic variants that affect drug responses is the main focus of pharmacogenetics (PGx). This research field is aimed at tailoring and predicting the efficacy of therapeutic regimens while reducing adverse drug events (toxicity) (Arbitrio et al., 2021) by using the individual genetic composition. PGx is important for precision medicine, given its role in personalizing therapeutic regimens and improving treatment outcomes. PGx testing is, according to evidence, cost-effective in clinical practice, either in public (Morris et al., 2022; Sukri et al., 2022) or private institutions. Therefore, PGx outcomes should be integrated into healthcare systems and public health policies. Research on this discipline is growing in Latin American countries, but it is still limited to some populations (Salas-Hernández et al., 2023). One of the characteristics of the Latin American population is heterogeneity, with variable levels of genetic admixture from African, European, and Amerindian ancestries (Bonifaz-Peña et al., 2014; Suarez-Kurtz and Parra, 2018). This makes extrapolation of available PGx data from other populations not possible and reinforces the need to properly characterize each population in terms of genetic variants with clinical consequences.

Dominican Republic (DR) is the second largest and populated country in the Caribbean after Cuba, and it occupies two-thirds of the Hispaniola Island. DR has received immigrants from other Caribbean Islands (mainly from Haiti), West and East Asia, and Jewish populations from Europe (Keegan, 2013). Therefore, the genetic make-up of the DR population has been shaped by the immigration of Europeans and the trans-Atlantic slave trade. The estimation of ancestry in this admixed population is biased by history, renders epidemiological implication, and is the subject of exhaustive research (Bryc et al., 2010; Moreno-Estrada et al., 2013; Tishkoff et al., 2009). In addition, mtDNA sequence data reveal an incredible degree of genetic variation within the DR population (Oakley et al., 2017). The high rate of inbreeding in this population may increase the frequency of recessive variants and support the identification of rare variants associated with late-onset Alzheimer disease (Vardarajan et al., 2015). It has also been suggested that this population has distinct risk alleles that contribute to systemic lupus erythematosus (SLE), lupus nephritis, and neuropsychiatric SLE susceptibility (Liu et al., 2019).

However, the frequencies of clinically relevant pharmacogenetic variants in the DR population have been poorly characterized. The first step toward the implementation of PGx tools in admixed populations such as DR requires accurate information on PGx allele frequencies in the general population. Increasing understanding of the PGx allele and phenotype frequencies distribution provides useful information as a reference to support clinical application (e.g., drug dosing guidelines and annotated drug labels) and as guidance for future PGx studies and clinical genetic testing panel designs (Daly, 2017).

The cytochrome P450 (CYP) superfamily and transporter proteins encoded by the *ABCB1* and *SLC45A2* genes play a critical role in the biotransformation of xenobiotics and endogenous compounds. Other genes such as *AHR* and *EPHX1* and genes related to DNA damage repair (*XRCC1, ERCC*, and *MGMT*) also encode proteins that are part of signaling pathways and participate in the metabolism of xenobiotics, thus influencing toxicity and mutagenesis (Liu et al., 2016). The expression of these genes is regulated by genetic, pathophysiological, environmental, and nutritional factors, and their hepatic levels vary among individuals within a population. Polymorphic variants may alter the functioning of metabolic pathways that eliminate xenobiotics, including drugs (Waring, 2020). Less efficient repair capacity may increase the risk of developing cancer. It also decreases the removal of cytostatic-induced adducts in DNA at the tumor cell level, which correlates with better clinical response and survival (Ma et al., 2018). Therefore, these systems are relevant to personalized medicine. They confer inter-individual differences in clinical effects of commonly used drugs, like proton pump inhibitors (El Rouby et al., 2018), analgesic, antihypertensives, antidepressants (Nofziger et al., 2020), antiepileptic (Fricke-Galindo et al., 2018), anticoagulants (Raymond et al., 2021), and antitumor drugs (Bosch et al., 2006), among others. Polymorphic variants are also considered genetic markers for individual prognosis in the therapeutic response (Balkan et al., 2020).

In this study, the frequencies of clinically relevant PGx traits in the Dominican Republic population were characterized. Thirty-two single-nucleotide variants (SNVs) from 14 genes that influence drug metabolism were genotyped. This knowledge may be used as a reference to support the implementation of personalized medicine approaches for disease prevention and pharmacogenetic testing in public health policies.

## 2 Materials and methods

### 2.1 General description of the population sample

This is a cross-sectional study in which the presence of SNVs was evaluated in 150 recruited participants. They were healthy volunteers living in Santo Domingo, Dominican Republic, and they were recruited at the “*Centro Médico de Diabetes, Obesidad, y Especialidades*” (CEMDOE) and the “*Instituto Tecnológico de Santo Domingo*” (INTEC). All participants were older than 18 years, with the average body mass index of 25.8, and they gave informed consent for inclusion. The main recorded demographic variable was skin color by self-classification, and individuals were grouped in three categories: White, admixed, and Black.

### 2.2 Genotyping and ancestry analysis

Whole-blood samples were obtained by venipuncture, and genomic DNA extraction was performed by using a QIAGEN DNeasy^®^ Blood and Tissue Kit, following the manufacturer’s recommendations. A total of 32 SNVs present in 14 genes (*CYP2D6, CYP2C9, CYP2C8, CYP2C19, CYP3A4, CYP3A5, CYP1A1, AHR, ABCB1, SLC45A2, XRCC1, XRCC3, ERCC2*, and *MGMT*) and 28 ancestry informative markers (Phillips et al., 2012) were determined by targeted sequencing (amplicon sequencing method) on an Illumina NovaSeq/HiSeq PE150 platform (Illumina, Inc., San Diego, CA, United States), according to Illumina protocols.

Ancestry informative markers (AIMs) were selected from a previous publication (Kosoy et al., 2009). The program STRUCTURE v 2.3.4 was used to estimate individual global ancestry proportions (Pritchard et al., 2000). The parameters of the program were set to run 200,000 Markov Chain Monte Carlo steps after a burn-in period of length 100,000 with 20 replicates for a K-value of 3. Putative population origin of samples and the admixture model with correlated allele frequencies among populations were used. Alignment of the replicates was performed by the computer program CLUMPP Version 1.1.2 (Jakobsson and Rosenberg, 2007).

Genotypes from parental populations were extracted from the Human Genome Diversity Project (HGDP) (Bergström et al., 2020) and the 1000 Genomes Project (Fairley et al., 2019) datasets. Parental populations from the 1000 Genomes Project comprised 107 Europeans (IBS Iberians) and 405 Africans (YRI Yoruba, ESN Nigeria, MSL Sierra Leona, and GWD Gambia). HGDP populations consisted of 60 Spanish, 35 Mexican (Pima and Maya), 22 Brazilian (Karitiana and Suri), and seven Colombian (Colombia) genotypes. SNV data were extracted using the online tool SPSmart SNPforID 34-plex variability browser (http://spsmart.cesga.es/snpforid.php) (Amigo et al., 2008).

### 2.3 Statistical analysis

Wright’s F_ST_ statistic was used as a metric to quantify genetic differentiation at SNVs across populations and within the sample of the Dominican population. Pairwise variant-specific F_ST_ values were calculated as SNV-specific F_ST_ = [(p1−p2)^∧^2/((p1+p2)(2-p1-p2)], where p1 and p2 denote the frequencies of a given allele in population 1 and population 2, respectively (Chen et al., 2010). According to these authors, an F_ST_ value lesser than 0.05 should reveal low genetic divergence, a value from 0.05 to 0.15 was identified as having moderate divergence, F_ST_ values from 0.15 to 0.25 revealed large genetic divergence, and values over 0.25 were indicative of very large divergence.

Allelic and genotype frequencies were calculated. The chi-squared (χ2) test was used to check Hardy–Weinberg equilibrium (HWE), and the Fisher exact test (Fisher–Freeman–Halton format) (Freeman and Halton, 1951) was used to compare genotype distribution among subgroups. Statistical differences were evaluated by means of Kruskal–Wallis (KW) analysis of variance, followed by *post hoc* Dunn’s test using a Bonferroni corrected alpha of 0.017 for multiple comparisons. Two-tailed tests were used, and statistical significance was set at *p*-value < 0.05. Statistical analyses were performed using RStudio Programming Environment for Data Analysis (Team, 2023) and ‘stats’ package version 4.3.2.

## 3 Results

### 3.1 Prevalence of target variants in pharmacogenes

Genotype and allele frequencies were calculated for the selected SNVs present in metabolic and DNA repair genes (Table 1). All evaluated SNVs but seven were in HWE, and the exceptions were rs12769205 and rs4244285 from *CYP2C19* and rs16947, rs3892097, rs1058164, rs1135840, and rs28371725 from *CYP2D6*. The reference genotype was the most common one, and it was observed in 21 variants, followed by the heterozygous genotype, which was observed in just seven SNVs. An alternative homozygous genotype was more prevalent in only four SNVs, and in general, they showed low frequency (≤10.0%) within the analyzed sample. Moreover, some other exceptions were the following: rs169447, rs1058164, and rs1135840 from *CYP2D6*; rs3758581 from *CYP2C19*; rs776746 from *CYP3A5*; rs2242480 and rs2740574 from *CYP3A4*; rs1045642 and rs2032582 from *ABCB1*; rs35395 from *SCL45A2*; rs25487 from *XRCC1*; and rs10764896 from *MGMT*. Population heterozygosity exhibited a wide range (1.3%–54.0%) among Dominicans, and it was the most common genotype for rs2242480 and rs2740574 from *CYP3A4*, rs776746 from *CYP3A5*, rs1045642 from *ABCB1*, rs35395 from *SLC45A2*, and rs10764896 from *MGMT*. The AC heterozygous genotype was the most frequent one (47.0%) for the rs2032582 tri-allelic SNV, followed by the CC alternative genotype (43.0%). The other genotypes were infrequent, and the TT genotype was not detected in the sample (Table 1).

**TABLE 1:** Genotype and allele frequencies of 32 SNVs in a sample from the DR population (N = 150).

SNV Ref > Alt	Genotypic frequency			Allele alternative frequency (95% CI)	HWE χ2 p-value
	Reference	Heterozygous	Alternative		
rs16947 G > A	0.45	0.35	0.19	0.37 (0.31─0.43)	0.00^a^
rs3892097 C > T	0.66	0.34	0.00	0.17 (0,13─0.21)	0.01^a^
rs1058164 C > G	0.21	0.37	0.41	0.60 (0.54─0.66)	0.01^a^
rs61736512 C > T	0.93	0.07	0.01	0.04 (0.02─0.06)	0.11
rs28371706 G > A	0.86	0.13	0.01	0.08 (0.05─0.11)	0.08
rs28371704 T > C	0.79	0.19	0.02	0.12 (0.08─0.15)	0.45
rs28371703 G > T	0.79	0.19	0.02	0.12 (0.08─0.15)	0.45
rs1065852 G > A	0.59	0.37	0.05	0.23 (0.18─0.28)	0.67
rs1135840 C > G	0.21	0.37	0.41	0.60 (0.54─0.66)	0.01^a^
rs59421388 C > T	0.93	0.07	0.01	0.04 (0.02─0.06)	0.11
rs28371725 C > T	0.93	0.05	0.01	0.04 (0.02─0.06)	0.11
rs12248560 C > T	0.63	0.35	0.03	0.20 (0.15─0.25)	0.31
rs12769205 A > G	0.63	0.37	0.00	0.18 (0.14─0.23)	0.01^a^
rs4244285 G > A	0.64	0.36	0.00	0.18 (0.14─0.22)	0.01^a^
rs3758581 A > G	0.00	0.07	0.93	0.97 (0.95─0.99)	0.67
rs1799853 C > T	0.79	0.20	0.01	0.11 (0.08─0.15)	0.95
rs1057910 A > C	0.93	0.07	0.00	0.03 (0.01─0.05)	0.67
rs10509681 T > C	0.83	0.17	0.01	0.09 (0.06─0.12)	0.83
rs11572103 T > A	0.83	0.17	0.01	0.09 (0.06─0.12)	0.83
rs776746 T > C	0.15	0.49	0.37	0.61 (0.55─0.67)	0.78
rs2242480 C > T	0.30	0.53	0.17	0.44 (0.38─0.49)	0.39
rs2740574 C > T	0.15	0.43	0.42	0.63 (0.58─0.69)	0.32
rs1048943 T > C	0.83	0.16	0.01	0.09 (0.06─0.13)	0.50
rs2066853 G > A	0.55	0.36	0.09	0.27 (0.22─0.32)	0.33
rs1045642 A > G	0.13	0.54	0.33	0.60 (0.55─0.66)	0.12
rs2032582A > C/(T)^b^	0.09	0.47/(0.01)	0.43/(0.0)	0.67 (0.62─0.73)	0.17
rs35395 T > C	0.21	0.51	0.29	0.54 (0.48─0.60)	0.81
rs25487 T > C	0.05	0.29	0.66	0.81 (0.76─0.85)	0.47
rs861539 G > A	0.49	0.42	0.09	0.30 (0.24─0.35)	0.94
rs13181 T > G	0.52	0.40	0.08	0.28 (0.23─0.33)	0.92
rs10764896 G > A	0.25	0.51	0.24	0.50 (0.44─0.55)	0.74
rs11016885 T > C	0.65	0.31	0.03	0.19 (0.14─0.24)	0.83

Alleles are reported in forward orientation; RefSeqGene database (O’Leary et al., 2016). 95% CI, 95% confidence interval; HWE, Hardy–Weinberg equilibrium; χ^2^p-value.

^a^ Indicates statistically significant deviation from HWE (p < 0.05).

^b^ Tri-allelic SNV.

All of the abovementioned SNVs but *CYP2D6* rs16947 and *MGMT* 10764896 were more frequent than the reference homozygous genotype. Meanwhile, the genotype frequencies of *CYP3A5* rs776746, rs2242480, *CYP3A4* rs2740574, *ABCB1* rs2032582, and *SLC45A2* rs35395 differed significantly (Fisher exact test, *p* < 0.05) among skin color subgroups (see Supplementary Table S1 for extended information on allele frequencies in the sample).

**FIGURE 1:**
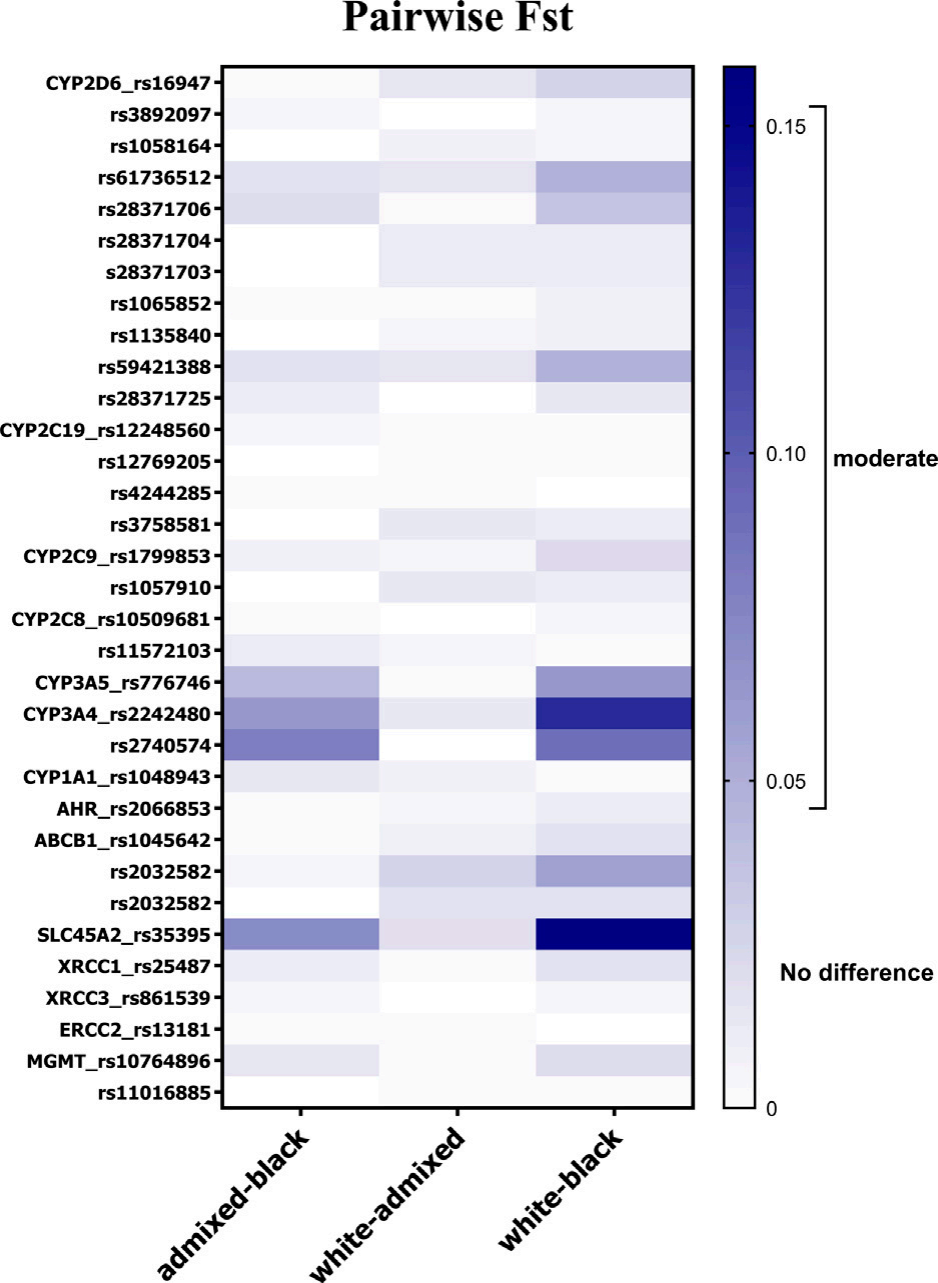
Allele-specific F_ST_ values for 32 SNVs in pairwise comparisons according to skin color in individuals from the DR cohort. Gradient bar F_ST_ scale: 0.05–0.15 indicates moderate divergence, 0.15–0.25 indicates large divergence, and over 0.25 indicates very large differentiation.

Genetic heterogeneity among skin color groups was further explored by means of F_ST_ statistic within the cohort stratified by skin color (Figure 1). The lowest genetic differentiation was observed between White and admixed individuals (mean F_ST_ = 0.007, SD: 0.007). None of the variants approached the moderate differentiation threshold. Although small, mean F_ST_ values of 0.026 (SD: 0.037) and 0.013 (SD: 0.021), respectively, indicated greater differentiation in Black vs. White and vs. admixed individuals. The variants in *CYP3A4* and *CYP3A5* showed moderate differentiation in Black vs. White and vs. admixed comparisons. *ABCB1* rs2032582 presented moderate divergence in Black vs. White comparison (F_ST_ = 0.058). Large differentiation was identified for *SLC45A2* rs35395 in Black vs. White individuals (F_ST_ = 0.159), and moderate differentiation was seen in Black vs. admixed individuals (F_ST_ = 0.072). rs61736512 and rs59421388 in *CYP2D6* gene (F_ST_ = 0.048) approached the F_ST_ threshold for moderate genetic divergence in White vs. Black individuals.

### 3.2 Genetic differentiation between DR and other populations

**FIGURE 2:**
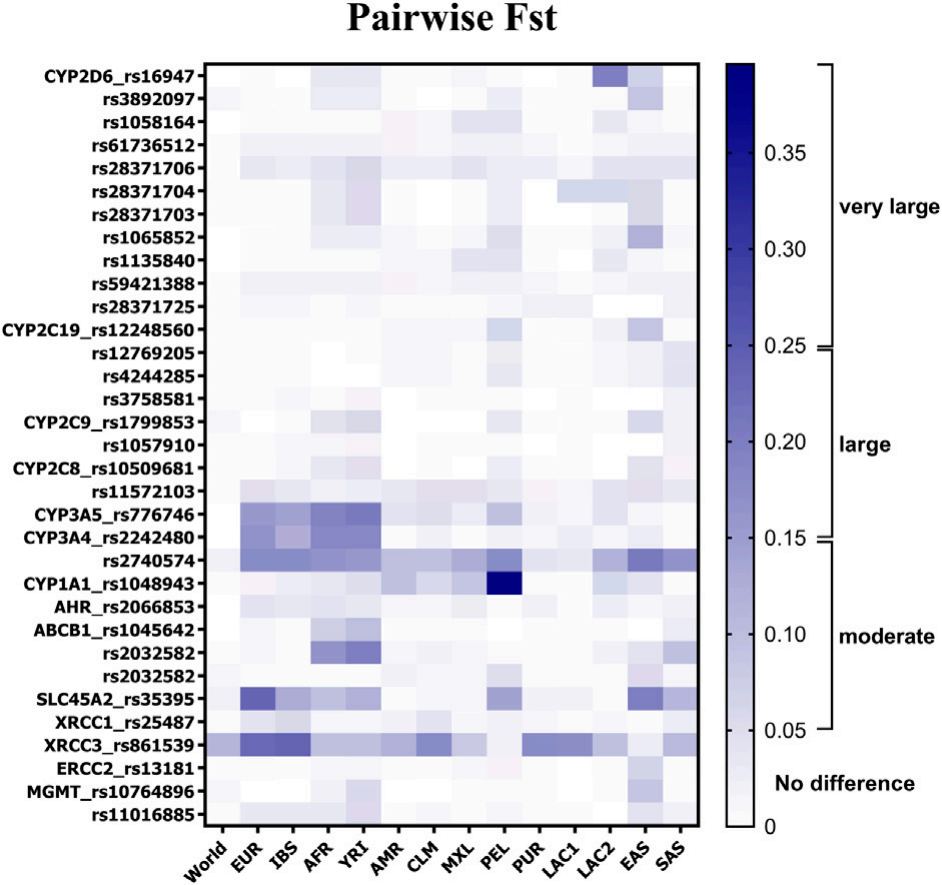
Allele-specific F_ST_ values for 32 SNVs in pairwise comparisons between DR and other populations. Gradient bar F_ST_ scale: 0.05–0.15 indicates moderate divergence, 0.15–0.25 indicates large divergence, and over 0.25 indicates very large differentiation. Frequency data were collected from the 1000 Genome Project database. EUR, Europeans; IBS, Iberians; AFR, Africans; YRI, Yorubas; AMR, Latin Americans; CLM, Colombians; MXL, Mexicans; PEL, Peruvians; PUR, Puerto Ricans; EAS, East Asians; SAS, South Asians; LAC1, Latin American individuals with Afro-Caribbean ancestry; LAC2, Latin American individuals with mostly European and Native American ancestries.

Pairwise F_ST_ analysis revealed that, on average, the DR cohort displayed high genetic similarity to Latin American populations (F_ST_ = 0.018, SD: 0.03) (Figure 2), specifically to Colombians (F_ST_ = 0.021, SD: 0.035), Mexicans (F_ST_ = 0.021; SD: 0.029), Puerto Ricans (F_ST_ = 0.013, SD: 0.031), and Latin Americans with Afro-Caribbean ancestry (LAC1, F_ST_ = 0.013, SD: 0.031). Values of F_ST_ lesser than 0.05 indicated that there was low genetic differentiation between these populations, given the selected SNVs. Although small, higher differences were identified among Dominicans and Europeans (F_ST_ = 0.040, SD: 0.069), Iberians (F_ST_ = 0.035, SD: 0.059), South Asians (F_ST_ = 0.029, SD: 0.037), and Latin American individuals with high European and Native American ancestries (LAC2, F_ST_ = 0.027, SD: 0.041). The average F_ST_ value was found to be near the threshold of moderate differentiation for pairwise comparisons between Dominicans and Yorubas (F_ST_ = 0.054, SD: 0.058), East Asians (F_ST_ = 0.049, SD: 0.05), Africans (F_ST_ = 0.046, SD: 0.055), and Peruvians (F_ST_ = 0.045, SD: 0.074). Extended information about the minor allele frequency (MAF) of the SNVs of Dominicans compared to other populations is presented in Supplementary Table S2.

Several SNVs in *CYP2D6* pharmacogenes exceeded the F_ST_ threshold of moderate genetic differentiation (Figure 2). As can be observed, the variants analyzed in *CYP2D6* (rs16947, rs3892097, rs28371706, rs28371704, rs28371703, and rs1065852) presented more genetic differentiation within the DR cohort when compared to East Asians and, in a lesser extent, to Yorubas and LAC2. Large divergence was observed just for rs16947 in comparison with LAC2 (F_ST_ = 0.195). Two SNVs in the *CYP2C* gene family exceeded the moderate differentiation threshold in pairwise comparisons: rs12248560 in DR vs. Peruvians (F_ST_ = 0.061) and in DR vs. East Asians (F_ST_ = 0.089), and rs1799853 in DR vs. Yorubas (F_ST_ = 0.06) and in DR vs. East Asians (F_ST_ = 0.059). On the other hand, the *CYP3A* gene family presented large genetic divergence (F_ST_ > 0.15) in comparisons between Dominicans and one of the following: Europeans, Iberians, Africans, and Yorubas. rs2740574 in the *CYP3A4* gene was the SNV with the highest genetic differentiation value within the DR cohort, which ranged from moderate-to-large differentiation values in most of the comparisons. Only Puerto Ricans (F_ST_ = 0.039) and LAC1 (F_ST_ = 0.034) showed low differentiation with Dominicans. Meanwhile, rs1048943 in the *CYP1A1* gene exhibited very large differentiation (>0.25) when compared to Peruvians and moderate differences compared to Latin Americans (F_ST_ = 0.095), Colombians (F_ST_ = 0.057), Mexicans (F_ST_ = 0.09), and LAC2 (F_ST_ = 0.062).

In the *ABCB1* gene, rs1045642 presented moderate differentiation and rs2032582 exhibited large differentiation, compared to Africans and Yorubas (Figure 2). The rs2032582 variant also revealed moderate differentiation with East Asians (F_ST_ = 0.055) and South Asians (F_ST_ = 0.096). For *SLC45A2* rs35395, large-to-moderate differentiation was found when compared to most of the populations (i.e., Europeans, Africans, East Asians, and South Asians). A high similarity to Latin Americans, including LAC1 and LAC2, was found, while Peruvians were an exceptional case, showing moderate divergence to DR individuals (F_ST_ = 0.141).

Regarding DNA repair genes, the *XRCC1* rs25487 variant showed moderate differences when compared to Iberians (F_ST_ = 0.06, Figure 2). Meanwhile, the *ERCC2* gene rs13181 SNV showed differences with populations of East Asians (F_ST_ = 0.068). Moderate genetic differentiation was found for both MGMT SNVs when compared to Yorubas, but only rs10764896 presented differences from East Asians (F_ST_ = 0.087). The largest variability across populations was observed for *XRCC3* rs861539. Differentiation values were observed when the cohort was pairwise compared to Europeans (F_ST_ = 0.231), Iberians (F_ST_ = 0.239), Colombians (F_ST_ = 0.18), Puerto Ricans (F_ST_ = 0.18), and LAC1 (F_ST_ = 0.173). The analysis considering East Asians (F_ST_ = 0.027) and Peruvians (F_ST_ = 0.022) was the only one that did not reach the moderate differentiation threshold.

In summary, most of the genetic differences between the Dominican population sample and the rest of the populations were found in six SNVs (Figure 2). They were rs776746, rs2740574, rs2242480, rs1048943, rs35395, and rs861539 since they presented more F_ST_ values in the moderate and large ranges than the rest of the SNVs under comparisons. On average, Dominicans exhibited large genetic similarities to Latin American populations, except for the Peruvians.

### 3.3 Admixture proportions in the Dominican sample

**FIGURE 3:**
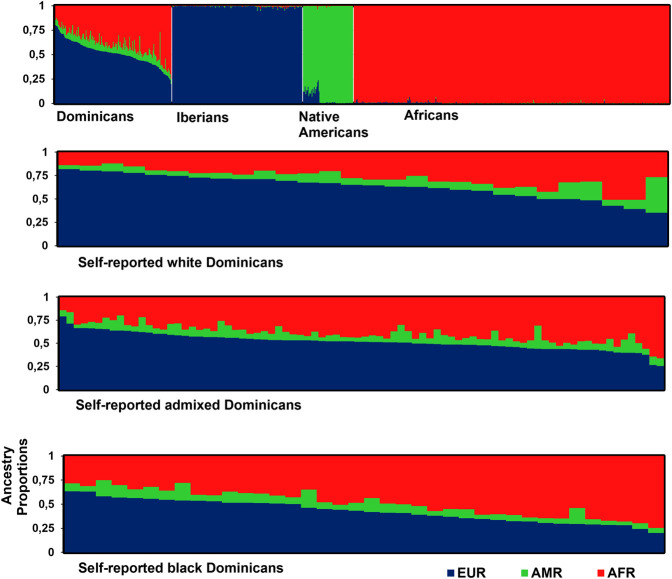
Individual ancestry estimates for different populations. White vertical lines separate populations. Each individual is represented by a vertical bar partitioned in three different colors, which indicate the individual’s estimated membership fractions in each cluster: blue for Europeans (EUR, Iberians), green for Amerindians (AMR, Native Americans), and red for Africans (AFR). Population data were collected from the HGDP-CEPH and 1000 Genome Project databases (n = 786).

The genetic structure of the DR urban population cohort revealed that, in average, admixture estimates of individuals were 51.6%, 39.5%, and 8.9%, respectively, for European, African, and Amerindian ancestries (Figure 3). The proportions of European ancestry decreased progressively from self-reported White 63.2% (35.2%–81.9%), admixed 51.8% (25.5%–79.2%), to Black individuals 42.8% (20.0%–63.4%) (KW: European, *p* < 0.001). The opposite trend was observed regarding African ancestry, which averaged 27.5% (12. 2%–51.0%), 39.4% (14.1%–66.3%), and 48.6% (25.1%–74.7%), respectively, in White, admixed, and Black individuals (KW: African, *p* < 0.001). The average Amerindian ancestry was homogeneous among the groups: 9.3% (4.1%–38.1%), 8.9% (3.1%–25.0%), and 8.6% (3.8%–18.9%) (KW: Amerindian, *p* = 0.927). The African and European components explained 90.1% of genetic diversity in the sample; therefore, they should mostly determine the frequency distribution of SNVs with pharmacological relevance.

## 4 Discussion

There are an increasing number of drugs that are currently required, or at least are recommended, by regulatory authorities to perform pharmacogenomics testing for preventing drug-related toxicity or improving drug efficacy (van der Wouden et al., 2022). This study reported the allelic variation in 32 SNVs from 14 genes involved in drug biotransformation, treatment outcome, and efficacy within a sample of 150 healthy volunteers living in Santo Domingo, Dominican Republic. Ancestry analysis revealed that the complex admixture landscape of the Dominican Republic influenced the distribution of the studied variants. Pairwise comparisons and admixture analyses suggested that drug responses may individually vary by skin color. Hence, the observed HWE deviations may be caused by population stratification, an element derived from DR’s population structure, which may have pharmacogenetic implications.

*CYP* P450 is the most important gene family that contributes to the oxidative metabolism of a wide range of drugs and endogenous compounds. Determinants of inter-individual variability in *CYP* enzyme activity include the presence of genetic variants that result in changes in enzyme activity. This, together with endogenous and environmental factors, accounts for the observed inter-ethnic variability in drug response, therapeutic effect, and adverse reactions (Daly, 2017). The *CYP2D6* gene is responsible for the metabolism of approximately 20% of the commonly used drugs, including tricyclic antidepressants, opioids, antiemetics, and antiarrhythmics, and it is a highly polymorphic gene. Therefore, its allele variants have been the focus of attention (Hicks et al., 2014). *CYP2D6* alleles are usually classified as having no function, decreased function, normal function, or increased function. Great variability in the *CYP2D6* allele frequencies has been reported for Hispanic Mestizo populations (López et al., 2005). The variants rs61736512 and rs59421388 of the *CYP2D6* gene approached the F_ST_ threshold for moderate genetic divergence in White vs. Black individuals, which means that important metabolic differences in individuals may be identified when skin color is taken into consideration. The rs61736512 and rs59421388 (both C > T, missense variants) variants define the core of several star alleles; both define alleles *29, *70, *149, *155, *156, and *157, while rs61736512 also defines allele *107 and rs59421388 defines allele *109. Those two variants are associated with the decreased metabolism of debrisoquine, according to PharmaGKB (Stojanović-Marković et al., 2022).

Meanwhile, *CYP2C9* is one of the most studied *CYPs* in pharmacogenetics since it also metabolizes a broad spectrum of commonly used drugs. Two common variant alleles, CYP2C9*2 rs1799853 and CYP2C9*3 rs1057910, code for proteins with single amino acid changes, resulting in a lower enzyme activity (Daly et al., 2017). It is well-established that, on average, individuals who carry one or two copies of these *CYP2C9* variant alleles require a lower dose of warfarin to achieve the international normalized ratio target value (Daly, 2013). African Americans have been associated with requiring lower warfarin doses as the *CYP2C9* variant alleles occurs less frequently in individuals of African ancestry (Cavallari et al., 2010). Its minor allele frequency ranges from 0.01 to 0.06 in African Americans, and accordingly, it was 0.03 in the Dominican sample, while MAFs in Puerto Ricans for CYP2C9*2 and CYP2C9*3 are 0.07 and 0.05, respectively (Claudio-Campos et al., 2015b). Studies performed by Villagra et al. informed that admixture may explain deviations from published findings regarding CYP2C9*2 allele frequencies in Puerto Ricans (Villagra et al., 2010). CYP2C9*2 and CYP2C9*3 variants ranged closely to reports in Brazilians (Suarez-Kurtz et al., 2012) and Venezuelans (Flores-Gutiérrez et al., 2017). In Cubans, a population similar to Dominicans in terms of ancestry history, individuals classified as Cuban Whites and Cuban Mestizos, show MAF values of 0.06–0.17 for CYP2C9*2 and 0.05–0.09 for CYP2C9*3, according to Rodrigues-Soares et al. (2020), or 0.07–0.13 for CYP2C9*2 and 0.01–0.05 for CYP2C9*3, according to Reyes-Reyes et al. (2024). As expected, these frequencies range very close to the ones observed here, which were 0.07–0.16 for White-DR and 0.02–0.12 for admixed-DR. The MAFs of CYP2C9*2 and CYP2C9*3 are lower in Mexicans living in Mexico (Mexican Mestizos and Mexican Tapehuana) than in Mexican Americans 0.06–0.08 (Claudio-Campos et al., 2015a) and in Dominicans 0.03–0.11. The CYP2C9*2 and *3 variants (most frequent among Caucasians) account for 15%–20% of the warfarin dose variability in various populations worldwide. These findings suggest that inter-individual variations in ancestral contribution may influence the allele frequencies and the response to warfarin in different Latin American populations.

CYP2C19 is a key enzyme involved in the metabolism of the antiplatelet drug clopidogrel, selective serotonin reuptake inhibitors, and proton pump inhibitors (Daly, 2017). The clinically most relevant variant alleles are CYP2C19*2 (rs4244285; 681G > A) and CYP2C19*3 (rs4986893; 636G > A) null alleles, which produce an inactive enzyme. CYP2C19*2 leads to a splicing defect, while CYP2C19*3 results in a premature stop codon and a truncated protein that lacks enzymatic activity. Homozygotes are poor metabolizers, who may benefit when using proton pump inhibitors since the plasma drug concentration is higher, resulting in better control of gastro-esophageal acidity. CYP2C19*3 is associated with poor metabolism of proguanil, a prophylactic antimalarial drug (Lee, 2013). Clopidogrel is also a prodrug, and *CYP2C19* makes an important contribution to the activation steps. Because of such evidence, in 2010, the FDA added a boxed warning to the clopidogrel label, stating that poor metabolizers of *CYP2C19* may not benefit from treatment with this drug, and CPIC guidelines recommend the use of alternative antiplatelet drugs such as prasugrel and ticagrelor in both poor metabolizers and those carrying one loss of the active allele (Scott et al., 2013). SNVs in *CYP2C19* have not been well-described for the Hispanic population. One of the highest frequencies of CYP2C19*2 and *3 is found in the Venezuela population, associated with their Amerindian component (Castro de Guerra et al., 2013). CYP2C19*3 is less frequent than CYP2C19*2 worldwide, and it is almost absent in Latin Americans (Suarez-Kurtz et al., 2012). In accordance to these reports, it was not detected in the Dominican sample analyzed here.

On the contrary, *CYP1A1* rs1048943 (CYP1A1*2C) is highly frequent in different Latin American populations, mainly those with a clear Amerindian ancestry, whereas in Africans and Europeans, its frequency is low (Pérez-Morales et al., 2008). Thus, the minor Amerindian component estimated for this gene in Dominicans compared to other Latin American populations may explain the moderate divergence to Latin American populations.

The CYP3A5*3 allele renders a lack of this enzyme due to interferences with RNA splicing. The worldwide allele distribution of rs776746 (CYP3A5*3) increases from 18% in Africans to 94% in European populations (Fairley et al., 2019). On average, the frequency of CYP3A5*3 is near 80% in Latin Americans, but it was lower in the present study (61%). Tacrolimus is widely used as an immunosuppressant in solid organ and hematopoietic stem cell transplant patients. It is well-established that individuals who express the cytochrome P450 *CYP3A5* require, on average, a higher dose of this drug to achieve the required plasma levels. Current recommendations from CPIC for tacrolimus dosing suggest that if *CYP3A5* genotype information is available, a starting dose 1.5–2 times higher than normal could be used (Birdwell et al., 2015). Meanwhile, rs2740574 (CYP3A4*1B), which is commonly found in African populations, due to a suggested selection factor against non-African populations involving vitamin D metabolism (Schirmer et al., 2006), had a frequency of 63% in the sample under study.

Frequency estimates of *AHR* rs2066853 are 15% in Mexicans (Pérez-Morales et al., 2011) and 8% in Brazilians (Abnet et al., 2007). Both frequencies are lower than the 27% found in this study. The calculated frequency of *ABCB1* rs1045642 in this study (37%, A allele) was intermediate to reported frequencies in Spanish (48%) and African Americans (16%), but it was similar to that in Brazilians (39%) (Scheiner et al., 2010) and Cubans (36.5%) (Rodeiro et al., 2022).

Here, we showed that there were significant differences in the frequencies of variant alleles in genes associated with treatment outcomes in Dominicans. This should contribute in introducing ethnic-specific genotype-to-phenotype correlations for therapeutic approaches in the Dominican Republic. The analysis considered the group stratifications according to skin color, which may aid in identifying associations between causative variants and drug response. Future studies similar to that presented here should be performed in a bigger sample from the Dominican population, including individuals from rural and other areas outside the capital city. This will certainly generate more useful information on the clinical application of the pharmacogenetics for the Dominican population. However, to the best of our knowledge, this was the largest study where important pharmacogenes have been characterized in a sample of DR population, supporting the development of precision medicine in the country.

## Data Availability

The data analyzed in this study was obtained from INTEC. The following licenses/restrictions apply: participants prefer that personal genomic data be shared upon request. Requests to access these datasets should be directed to Dr. Berniza Calderón at berniza.calderon@cemdoe.com.

## References

[B1] Abnet C. C. Fagundes R. B. Strickland P. T. Kamangar F. Roth M. J. Taylor P. R. (2007). The influence of genetic polymorphisms in Ahr, CYP1A1, CYP1A2, CYP1B1, GST M1, GST T1 and UGT1A1 on urine 1-hydroxypyrene glucuronide concentrations in healthy subjects from Rio Grande do Sul, Brazil. Carcinogenesis 28 (1), 112–117. 10.1093/carcin/bgl131 16864595

[B2] Amigo J. Phillips C. Lareu M. Carracedo Á. (2008). The SNP for ID browser: an online tool for query and display of frequency data from the SNP for ID project. Int. J. Leg. Med. 122 (5), 435–440. 10.1007/s00414-008-0233-7 18491122

[B3] Arbitrio M. Scionti F. Di Martino M. T. Caracciolo D. Pensabene L. Tassone P. (2021). Pharmacogenomics biomarker discovery and validation for translation in clinical practice. Clin. Transl. Sci. 14 (1), 113–119. 10.1111/cts.12869 33089968 PMC7877857

[B4] Balkan E. Bilici M. Gundogdu B. Aksungur N. Kara A. Yasar E. (2020). ERCC2 Lys751Gln rs13181 and XRCC2 Arg188His rs3218536 gene polymorphisms contribute to subsceptibility of colon, gastric, HCC, lung and prostate cancer. J. BUON 25 (1), 574–581. 32277685

[B5] Bergström A. McCarthy S. A. Hui R. Almarri M. A. Ayub Q. Danecek P. (2020). Insights into human genetic variation and population history from 929 diverse genomes. Science 367, eaay5012. 10.1126/science.aay5012 32193295 PMC7115999

[B6] Birdwell K. A. Decker B. Barbarino J. M. Peterson J. F. Stein C. M. Sadee W. (2015). Clinical Pharmacogenetics Implementation Consortium (CPIC) guidelines for CYP3A5 genotype and tacrolimus dosing. Clin. Pharmacol. and Ther. 98(1), 19–24. 10.1002/cpt.113 25801146 PMC4481158

[B7] Bonifaz-Peña V. Contreras A. V. Struchiner C. J. Roela R. A. Furuya-Mazzotti T. K. Chammas R. (2014). Exploring the distribution of genetic markers of pharmacogenomics relevance in Brazilian and Mexican populations. PloS one 9 (11), e112640. 10.1371/journal.pone.0112640 25419701 PMC4242606

[B8] Bosch T. M. Meijerman I. Beijnen J. H. Schellens J. H. (2006). Genetic polymorphisms of drug-metabolising enzymes and drug transporters in the chemotherapeutic treatment of cancer. Clin. Pharmacokinet. 45, 253–285. 10.2165/00003088-200645030-00003 16509759

[B9] Bryc K. Auton A. Nelson M. R. Oksenberg J. R. Hauser S. L. Williams S. (2010). Genome-wide patterns of population structure and admixture in West Africans and African Americans. Proc. Natl. Acad. Sci. 107 (2), 786–791. 10.1073/pnas.0909559107 20080753 PMC2818934

[B10] Castro de Guerra D. Flores S. Izaguirre M. H. (2013). Distribution of CYP2C19* 2 and CYP2C19* 3 polymorphisms in Venezuelan populations with different admixture. Ann. Hum. Biol. 40 (2), 197–200. 10.3109/03014460.2012.749946 23249123

[B11] Cavallari L. Langaee T. Momary K. Shapiro N. Nutescu E. Coty W. (2010). Genetic and clinical predictors of warfarin dose requirements in African Americans. Clin. Pharmacol. and Ther. 87 (4), 459–464. 10.1038/clpt.2009.223 20072124

[B12] Chen J. Teo Y. Y. Toh D. S. Sung C. (2010). Interethnic comparisons of important pharmacology genes using SNP databases: potential application to drug regulatory assessments. Pharmacogenomics 11 (8), 1077–1094. 10.2217/pgs.10.79 20712526

[B13] Claudio-Campos K. Duconge J. Cadilla C. L. Ruaño G. (2015a). Pharmacogenetics of drug-metabolizing enzymes in US Hispanics. J Drug metabolism personalized Ther. 30 (2), 87–105. 10.1515/dmdi-2014-0023 PMC4447600 25431893

[B14] Claudio-Campos K. Orengo-Mercado C. Renta J. Y. Peguero M. García R. Hernández G. (2015b). Pharmacogenetics of healthy volunteers in Puerto Rico. J Drug metabolism personalized Ther. 30 (4), 239–249. 10.1515/dmpt-2015-0021 PMC4768757 26501165

[B15] Daly A. K. (2013). Optimal dosing of warfarin and other coumarin anticoagulants: the role of genetic polymorphisms. Archives Toxicol. 87(3), 407–420. 10.1007/s00204-013-1013-9 23376975

[B16] Daly A. K. (2017). Pharmacogenetics: a general review on progress to date. Br. Med. Bull. 124 (1), 65–79. 10.1093/bmb/ldx035 29040422

[B17] Daly A. K. Rettie A. E. Fowler D. M. Miners J. O. (2017). Pharmacogenomics of CYP2C9: functional and clinical considerations. J. personalized Med. 8(1), 1. 10.3390/jpm8010001 PMC5872075 29283396

[B18] El Rouby N. Lima J. J. Johnson J. A. (2018). Proton pump inhibitors: from CYP2C19 pharmacogenetics to precision medicine. Expert Opin. drug metabolism and Toxicol. 14 (4), 447–460. 10.1080/17425255.2018.1461835 PMC5942154 29620484

[B19] Fairley S. Lowy-Gallego E. Perry E. Flicek P. (2019). The International Genome Sample Resource (IGSR) collection of open human genomic variation resources. Nucleic acids Res. 48 (D1), D941-D947–D947. 10.1093/nar/gkz836 PMC6943028 31584097

[B20] Flores-Gutiérrez S. Rodríguez-Larralde Á. Vivenes de Lugo M. Castro de Guerra D. (2017). Distribution of polymorphisms in the CYP2C9 gene and CYP2C19/CYP2C9 haplotypes among Venezuelan populations. Ann. Hum. Biol. 44 (2), 191–198. 10.1080/03014460.2016.1192218 27230833

[B21] Freeman G. Halton J. H. (1951). Note on an exact treatment of contingency, goodness of fit and other problems of significance. Biometrika 38 (1/2), 141–149. 10.2307/2332323 14848119

[B22] Fricke-Galindo I. Jung-Cook H. Llerena A. López-López M. (2018). Pharmacogenetics of adverse reactions to antiepileptic drugs. Neurol. Engl. Ed. 33 (3), 165–176. 10.1016/j.nrl.2015.03.005 25976948

[B23] Hicks J. K. J Swen J. Gaedigk A. (2014) Challenges in CYP2D6 phenotype assignment from genotype data: a critical assessment and call for standardization. Curr. Drug Metab. 15, 218–232. 10.2174/1389200215666140202215316 24524666

[B24] Jakobsson M. Rosenberg N. A. (2007). CLUMPP: a cluster matching and permutation program for dealing with label switching and multimodality in analysis of population structure. J. Bioinforma. 23 (14), 1801–1806. 10.1093/bioinformatics/btm233 17485429

[B25] Keegan W. (2013). Caribbean Islands: archeology, 1. John Wiley and Sons, Ltd.

[B26] Kosoy R. Nassir R. Tian C. White P. A. Butler L. M. Silva G. (2009). Ancestry informative marker sets for determining continental origin and admixture proportions in common populations in America. Hum. Mutat. 30(1), 69–78. 10.1002/humu.20822 18683858 PMC3073397

[B27] Lee S.-J. (2013). Clinical application of CYP2C19 pharmacogenetics toward more personalized medicine. Front. Genet. 3, 318. 10.3389/fgene.2012.00318 23378847 PMC3561709

[B28] Liu H. Li J. Ye B. (2016). Correlation between gene polymorphisms of CYP1A1, GSTP1, ERCC2, XRCC1, and XRCC3 and susceptibility to lung cancer. Genet. Mol. Res. 15 (4). 10.4238/gmr15048813 27819744

[B29] Liu Z. Yu Y. Yue Y. Hearth-Holmes M. Lopez P. D. Tineo C. (2019). Genetic alleles associated with SLE susceptibility and clinical manifestations in Hispanic patients from the Dominican Republic. Curr. Mol. Med. 19 (3), 164–171. 10.2174/1566524019666190424130809 31032751

[B30] López M. Guerrero J. Jung–Cook H. Alonso M. E. (2005). CYP2D6 genotype and phenotype determination in a Mexican Mestizo population. Eur. J. Clin. Pharmacol. 61, 749–754. 10.1007/s00228-005-0038-2 16249913

[B31] Ma J. Setton J. Lee N. Y. Riaz N. Powell S. N. (2018). The therapeutic significance of mutational signatures from DNA repair deficiency in cancer. Nat. Commun. 9 (1), 3292. 10.1038/s41467-018-05228-y 30120226 PMC6098043

[B32] Moreno-Estrada A. Gravel S. Zakharia F. McCauley J. L. Byrnes J. K. Gignoux C. R. (2013). Reconstructing the population genetic history of the Caribbean. PLoS Genet. 9 (11), e1003925. 10.1371/journal.pgen.1003925 24244192 PMC3828151

[B33] Morris S. A. Alsaidi A. T. Verbyla A. Cruz A. Macfarlane C. Bauer J. (2022). Cost effectiveness of pharmacogenetic testing for drugs with Clinical Pharmacogenetics Implementation Consortium (CPIC) guidelines: a systematic review. Clin. Pharmacol. and Ther. 112 (6), 1318–1328. 10.1002/cpt.2754 36149409 PMC9828439

[B34] Nofziger C. Turner A. J. Sangkuhl K. Whirl‐Carrillo M. Agúndez J. A. Black J. L. (2020). PharmVar genefocus: CYP2D6. Clin. Pharmacol. and Ther. 107 (1), 154–170. 10.1002/cpt.1643 31544239 PMC6925641

[B35] Oakley E. R. Paulino-Ramírez R. Vega B. Vilar M. G. Mencía-Ripley A. Guerrero-Martínez S. A. (2017). Genetic diversity in the Dominican republic: implications for the population and demographic history of Hispaniola [poster] the 86th annual meeting of the American association of physical anthropologists (2017),. Available at: https://cris.unibe.edu.do/handle/123456789/36.

[B58] O’Leary N. A. Wright M. W. Brister J. R. Ciufo S. Haddad D. McVeigh R. (2016). Reference sequence (RefSeq) database at NCBI: current status, taxonomic expansion, and functional annotation. Nucleic Acids Res. 44 (D1), D733–D745. 10.1093/nar/gkv1189 26553804 PMC4702849

[B36] Pérez-Morales R. Castro-Hernández C. Gonsebatt M. E. Rubio J. (2008). Polymorphism of CYP1A1* 2C, GSTM1* 0, and GSTT1* 0 in a Mexican Mestizo population: a similitude analysis. Hum. Biol. 80 (4), 457–465. 10.3378/1534-6617-80.4.457 19317600

[B37] Pérez-Morales R. Méndez-Ramírez I. Castro-Hernández C. Martínez-Ramírez O. C. Gonsebatt M. E. Rubio J. (2011). Polymorphisms associated with the risk of lung cancer in a healthy Mexican Mestizo population: application of the additive model for cancer. Genet. Mol. Biol. 34 (4), 546–552. 10.1590/S1415-47572011005000053 22215955 PMC3229106

[B38] Phillips C. Fondevila M. Lareau M. V. (2012). A 34-plex autosomal SNP single base extension assay for ancestry investigations. Methods Mol. Biol. 830, 109–126. 10.1007/978-1-61779-461-2_8 22139656

[B39] Pritchard J. K. Stephens M. Donnelly P. (2000). Inference of population structure using multilocus genotype data. Genetics 155 (2), 945–959. 10.1093/genetics/155.2.945 10835412 PMC1461096

[B40] Raymond J. Imbert L. Cousin T. Duflot T. Varin R. Wils J. (2021). Pharmacogenetics of direct oral anticoagulants: a systematic review. J. personalized Med. 11 (1), 37. 10.3390/jpm11010037 PMC7826504 33440670

[B41] Reyes-Reyes E. Herrera-Isidrón J. A. Cuétara-Lugo E. Shkedy Z. Valkenborg D. Pérez-Novo C. A. (2024). Prevalence of single-nucleotide variants in twenty-five pharmacogenes from a Cuban sample cohort. Front. Pharmacol. 15, 1467036. 10.3389/fphar.2024.1467036 39403135 PMC11472837

[B42] Rodeiro I. Herrea J. Cuétara E. Garrido G. Reyes E. Martínez I. (2022). Prevalence of ABCB1 3435C> T polymorphism in the Cuban population. Drug metabolism personalized Ther. 37 (2), 141–148. 10.1515/dmpt-2020-0156 34860473

[B43] Rodrigues‐Soares F. Peñas‐Lledó E. M. Tarazona‐Santos E. Sosa‐Macías M. Terán E. López‐López M. (2020). Genomic ancestry, CYP 2D6, CYP 2C9, and CYP 2C19 among Latin Americans. Clin. Pharmacol. and Ther. 107 (1), 257–268. 10.1002/cpt.1598 31376146

[B44] Salas-Hernández A. López-Cortés A. Redal M. A. Fonseca-Mendoza D. Esperón P. González-Martínez F. (2023). An updated examination of the perception of barriers for pharmacogenomics implementation and the usefulness of drug/gene pairs in Latin America and the Caribbean. Front. Pharmacol. 14, 1175737. 10.3389/fphar.2023.1175737 37251329 PMC10213898

[B45] Scheiner M. A. M. Damasceno A. M. Maia R. C. (2010). ABCB1 single nucleotide polymorphisms in the Brazilian population. Mol. Biol. Rep. 37 (1), 111–118. 10.1007/s11033-009-9547-x 19437139

[B46] Schirmer M. Toliat M. R. Haberl M. Suk A. Kamdem L. K. Klein K. (2006). Genetic signature consistent with selection against the CYP3A4* 1B allele in non-African populations. Pharmacogenetics genomics 16 (1), 59–71. 10.1097/01.fpc.0000182779.03180.ba 16344723

[B47] Scott S. Sangkuhl K. Stein C. Hulot J. S. Mega J. Roden D. (2013). Clinical Pharmacogenetics Implementation Consortium guidelines for CYP2C19 genotype and clopidogrel therapy: 2013 update. Clin. Pharmacol. and Ther. 94(3), 317–323. 10.1038/clpt.2013.105 23698643 PMC3748366

[B48] Stojanović-Marković A. Zajc Petranović M. Škarić-Jurić T. Celinšćak Ž. Šetinc M. Tomas Ž. (2022). Relevance of CYP2D6 gene variants in population genetic differentiation. Pharmaceutics 14 (11), 2481. 10.3390/pharmaceutics14112481 36432672 PMC9694252

[B49] Suarez-Kurtz G. Genro J. De Moraes M. Ojopi E. Pena S. Perini J. (2012). Global pharmacogenomics: impact of population diversity on the distribution of polymorphisms in the CYP2C cluster among Brazilians. pharmacogenomics J. 12 (3), 267–276. 10.1038/tpj.2010.89 21173785

[B50] Suarez-Kurtz G. Parra E. (2018). Population diversity in pharmacogenetics: a Latin American perspective. J Adv. Pharmacol. 83, 133–154. 10.1016/bs.apha.2018.02.001 29801573

[B51] Sukri A. Salleh M. Z. Masimirembwa C. Teh L. K. (2022). A systematic review on the cost effectiveness of pharmacogenomics in developing countries: implementation challenges. pharmacogenomics J. 22 (3), 147–159. 10.1038/s41397-022-00272-w 35319010

[B52] Team R. C. (2023). R: a language and environment for statistical computing. Vienna, Austria: R Foundation for Statistical Computing. Available at: http://www.R-project.org/.

[B53] Tishkoff S. A. Reed F. A. Friedlaender F. R. Ehret C. Ranciaro A. Froment A. (2009). The genetic structure and history of Africans and African Americans. Science 324 (5930), 1035–1044. 10.1126/science.1172257 19407144 PMC2947357

[B54] van der Wouden C. H. Marck H. Guchelaar H.-J. Swen J. J. Van Den Hout W. B. (2022). Cost-Effectiveness of pharmacogenomics-guided prescribing to prevent gene-drug-related deaths: a decision-analytic model. Front. Pharmacol. 13, 918493. 10.3389/fphar.2022.918493 36120299 PMC9477094

[B55] Vardarajan B. N. Schaid D. J. Reitz C. Lantigua R. Medrano M. Jiménez-Velázquez I. Z. (2015). Inbreeding among caribbean hispanics from the Dominican republic and its effects on risk of alzheimer disease. Genet. Med. 17 (8), 639–643. 10.1038/gim.2014.161 25394174 PMC4430451

[B56] Villagra D. Duconge J. Windemuth A. Cadilla C. L. Kocherla M. Gorowski K. (2010). CYP2C9 and VKORC1 genotypes in Puerto Ricans: a case for admixture-matching in clinical pharmacogenetic studies. Clin. Chim. Acta 411 (17-18), 1306–1311. 10.1016/j.cca.2010.05.021 20488169 PMC2903218

[B57] Waring R. H. (2020). Cytochrome P450: genotype to phenotype. Xenobiotica 50 (1), 9–18. 10.1080/00498254.2019.1648911 31411087

