# Supplementary material for: Allele and genotype frequencies of variants in P450 cytochromes, transports, and DNA repair enzymes in the Dominican Republic population
Source: Front Pharmacol. 2025 Mar 5;15:1494482. doi: 10.3389/fphar.2024.1494482 (PMC11919896; doi:10.3389/fphar.2024.1494482)
Supplement: Supplementary file 1 [file Table1.docx]

| **Supplementary Table 1. Alternative allele frequencies of the studied loci in the Dominican Republic (DR) sample and stratified by skin color.** | | | | |  |
| --- | --- | --- | --- | --- | --- |
| **gene_SNP_alleles** | **General population** | **White DR** | **Admixed DR** | **Black DR** | *** p-value** |
| *CYP2D6*_rs16947_G>A | 0.37 | 0.27 | 0.38 | 0.42 | 0.45 |
| *CYP2D6*_rs3892097_C>T | 0.17 | 0.18 | 0.18 | 0.13 | 0.55 |
| *CYP2D6*_rs1058164_C>G | 0.6 | 0.54 | 0.62 | 0.61 | 0.10 |
| *CYP2D6*_rs61736512_C>T | 0.04 | 0.00 | 0.03 | 0.09 | 0.07 |
| *CYP2D6*_rs28371706_G>A | 0.08 | 0.04 | 0.06 | 0.14 | 0.13 |
| *CYP2D6*_rs28371704_T>C | 0.12 | 0.18 | 0.10 | 0.11 | 0.07 |
| *CYP2D6*_rs28371703_G>T | 0.12 | 0.18 | 0.10 | 0.11 | 0.07 |
| *CYP2D6*_rs1065852_G>A | 0.23 | 0.27 | 0.23 | 0.20 | 0.69 |
| *CYP2D6*_rs1135840_C>G | 0.6 | 0.54 | 0.61 | 0.62 | 0.20 |
| *CYP2D6*_rs59421388_C>T | 0.04 | 0.00 | 0.03 | 0.09 | 0.07 |
| *CYP2D6*_rs28371725_C>T | 0.04 | 0.05 | 0.05 | 0.01 | 0.64 |
| *CYP2C19*_rs12248560_ C>T | 0.2 | 0.21 | 0.18 | 0.24 | 0.73 |
| *CYP2C19*_rs12769205_A>G | 0.18 | 0.16 | 0.19 | 0.18 | 0.87 |
| *CYP2C19*_rs4244285_G>A | 0.18 | 0.16 | 0.19 | 0.17 | 0.85 |
| *CYP2C19*_rs3758581_A>G | 0.97 | 0.93 | 0.98 | 0.97 | 0.24 |
| *CYP2C9*_rs1799853_C>T | 0.11 | 0.16 | 0.12 | 0.07 | 0.30 |
| *CYP2C9*_rs1057910_A>C | 0.03 | 0.07 | 0.02 | 0.03 | 0.24 |
| *CYP2C8*_rs10509681_T>C | 0.09 | 0.11 | 0.10 | 0.07 | 0.81 |
| *CYP2C8*_rs11572103_T>A | 0.09 | 0.11 | 0.07 | 0.13 | 0.27 |
| *CYP3A5*_rs776746_T>C | 0.61 | 0.70 | 0.65 | 0.45 | **0.01** |
| *CYP3A4*_rs2242480_C>T | 0.44 | 0.29 | 0.39 | 0.64 | **<0.01** |
| *CYP3A4*_rs2740574_C>T | 0.63 | 0.71 | 0.70 | 0.42 | **<0.01** |
| *CYP1A1*_rs1048943_T>C | 0.09 | 0.07 | 0.12 | 0.05 | 0.63 |
| *AHR*_rs2066853_G>A | 0.27 | 0.21 | 0.27 | 0.30 | 0.85 |
| *ABCB1*_rs1045642_A>G | 0.60 | 0.52 | 0.61 | 0.64 | 0.59 |
| *ABCB1*_rs2032582_A>C | 0.67 | 0.52 | 0.68 | 0.75 | **0.04** |
| *ABCB1*_rs2032582_A>T | 0.01 | 0.04 | 0.00 | 0.00 | **0.03** |
| *SLC45A2*_rs35395_T>C | 0.54 | 0.71 | 0.58 | 0.32 | **<0.01** |
| *XRCC1*_rs25487_T>C | 0.81 | 0.77 | 0.79 | 0.87 | 0.60 |
| *XRCC3*_rs861539_G>A | 0.01 | 0.30 | 0.32 | 0.25 | 0.57 |
| *ERCC2*_rs13181_T>G | 0.28 | 0.25 | 0.30 | 0.26 | 0.67 |
| *MGMT*_rs10764896_G>A | 0.50 | 0.45 | 0.47 | 0.59 | 0.33 |
| *MGMT*_rs11016885_T>C | 0.19 | 0.16 | 0.20 | 0.20 | 0.92 |
| *p-value: Fisher exact test for differences among skin color categories in genotype frequencies, statistical differences are in bold. RD (N=150), White RD (N=28), Admixed RD (N=84), Black RD (N=38). | | | | | |

| **Supplementary Table 2.** **Alternative allele frequencies of the studied loci in the Dominican Republic (DR) sample compared to different populations.** | | | | | | | | | | | | | | | |
| --- | --- | --- | --- | --- | --- | --- | --- | --- | --- | --- | --- | --- | --- | --- | --- |
| **gene_SNP** | **DR** | **World (all)** | **EUR (all)** | **EUR (IBS)** | **AFR (all)** | **AFR (YRI)** | **AMR (all)** | **CLM** | **MXL** | **PEL** | **PUR** | **LAC1** | **LAC2** | **EAS** | **SAS** |
| *CYP2D6*  rs16947  G>A | 0.37 | 0.36 | 0.34 | 0.38 | 0.55 | 0.56 | 0.33 | 0.31 | 0.26 | 0.32 | 0.39 | 0.32 | 0.02 | 0.14 | 0.36 |
| *CYP2D6*  rs3892097  C>T | 0.17 | 0.09 | 0.19 | 0.15 | 0.06 | 0.06 | 0.13 | 0.17 | 0.13 | 0.07 | 0.15 | 0.14 | 0.11 | 0.002 | 0.11 |
| *CYP2D6*  rs1058164  C>G | 0.6 | 0.6 | 0.54 | 0.55 | 0.67 | 0.67 | 0.47 | 0.49 | 0.4 | 0.39 | 0.57 | 0.57 | 0.42 | 0.71 | 0.53 |
| *CYP2D6*  rs61736512  C>T | 0.04 | 0.03 | 0 | 0 | 0.11 | 0.11 | 0.003 | 0.005 | 0 | 0 | 0.005 | 0.03 | 0.005 | 0 | 0 |
| *CYP2D6*  rs28371706  G>A | 0.08 | 0.06 | 0.002 | 0.01 | 0.22 | 0.26 | 0.01 | 0.011 | 0 | 0.012 | 0.01 | 0.03 | 0 | 0 | 0 |
| *CYP2D6*  rs28371704  T>C | 0.12 | 0.07 | 0.17 | 0.14 | 0.02 | 0.005 | 0.1 | 0.12 | 0.1 | 0.03 | 0.13 | 0 | 0 | 0.001 | 0.08 |
| *CYP2D6*  rs28371703  G>T | 0.12 | 0.07 | 0.17 | 0.14 | 0.02 | 0.005 | 0.1 | 0.12 | 0.1 | 0.03 | 0.13 | 0.12 | 0.09 | 0.001 | 0.08 |
| *CYP2D6*  rs1065852  G>A | 0.23 | 0.24 | 0.2 | 0.17 | 0.11 | 0.11 | 0.15 | 0.19 | 0.15 | 0.07 | 0.18 | 0.17 | 0.12 | 0.57 | 0.16 |
| *CYP2D6*  rs1135840  C>G | 0.6 | 0.6 | 0.55 | 0.55 | 0.68 | 0.68 | 0.48 | 0.5 | 0.4 | 0.39 | 0.57 | 0.58 | 0.42 | 0.7 | 0.53 |
| *CYP2D6*  rs59421388  C>T | 0.04 | 0.03 | 0 | 0 | 0.11 | 0.11 | 0.003 | 0.005 | 0 | 0 | 0.005 | 0.02 | 0.005 | 0 | 0 |
| *CYP2D6*  rs28371725  C>T | 0.04 | 0.06 | 0.09 | 0.09 | 0.02 | 0.01 | 0.06 | 0.08 | 0.02 | 0.01 | 0.12 | 0.12 | 0.04 | 0.04 | 0.12 |
| *CYP2C19*  rs12248560  C>T | 0.2 | 0.15 | 0.22 | 0.22 | 0.24 | 0.25 | 0.12 | 0.13 | 0.12 | 0.04 | 0.18 | 0.16 | 0.1 | 0.02 | 0.14 |
| *CYP2C19*  rs12769205  A>G | 0.18 | 0.23 | 0.15 | 0.15 | 0.2 | 0.22 | 0.11 | 0.11 | 0.13 | 0.07 | 0.13 | 0.16 | 0.1 | 0.31 | 0.36 |
| *CYP2C19*  rs4244285  G>A | 0.18 | 0.22 | 0.15 | 0.15 | 0.17 | 0.17 | 0.11 | 0.11 | 0.13 | 0.06 | 0.13 | 0.16 | 0.11 | 0.31 | 0.36 |
| *CYP2C19*  rs3758581  A>G | 0.97 | 0.95 | 0.93 | 0.92 | 0.99 | 1 | 0.97 | 0.94 | 0.98 | 0.99 | 0.97 | 0.95 | 0.96 | 0.96 | 0.89 |
| *CYP2C9*  rs1799853  C>T | 0.11 | 0.05 | 0.12 | 0.14 | 0.01 | 0 | 0.1 | 0.12 | 0.1 | 0.02 | 0.14 | 0.13 | 0.08 | 0.001 | 0.04 |
| *CYP2C9*  rs1057910  A>C | 0.03 | 0.05 | 0.07 | 0.08 | 0.002 | 0 | 0.04 | 0.06 | 0.02 | 0.01 | 0.04 | 0.06 | 0.04 | 0.03 | 0.11 |
| *CYP2C8*  rs10509681  T>C | 0.09 | 0.05 | 0.12 | 0.15 | 0.01 | 0 | 0.1 | 0.12 | 0.1 | 0.02 | 0.14 | 0.11 | 0.08 | 0.001 | 0.03 |
| *CYP2C8*  rs11572103  T>A | 0.09 | 0.05 | 0 | 0.01 | 0.19 | 0.2 | 0.01 | 0 | 0 | 0.01 | 0.03 | 0.04 | 0.002 | 0 | 0.01 |
| *CYP3A5*  rs776746  T>C | 0.61 | 0.62 | 0.94 | 0.93 | 0.18 | 0.17 | 0.8 | 0.81 | 0.77 | 0.88 | 0.74 | 0.71 | 0.8 | 0.71 | 0.67 |
| *CYP3A4*  rs2242480  C>T | 0.44 | 0.42 | 0.08 | 0.12 | 0.85 | 0.85 | 0.39 | 0.29 | 0.39 | 0.58 | 0.34 | 0.28 | 0.34 | 0.27 | 0.37 |
| *CYP3A4*  rs2740574  C>T | 0.63 | 0.77 | 0.97 | 0.97 | 0.23 | 0.24 | 0.9 | 0.9 | 0.93 | 0.97 | 0.81 | 0.8 | 0.92 | 0.99 | 0.96 |
| *CYP1A1*  rs1048943  T>C | 0.09 | 0.13 | 0.03 | 0.02 | 0.01 | 0 | 0.35 | 0.28 | 0.34 | 0.71 | 0.14 | 0.08 | 0.29 | 0.25 | 0.13 |
| *AHR*  rs2066853  G>A | 0.27 | 0.27 | 0.11 | 0.12 | 0.46 | 0.45 | 0.17 | 0.18 | 0.13 | 0.22 | 0.15 | 0.24 | 0.14 | 0.37 | 0.15 |
| *ABCB1*  rs1045642  A>G | 0.60 | 0.6 | 0.48 | 0.54 | 0.85 | 0.88 | 0.57 | 0.56 | 0.52 | 0.62 | 0.57 | 0.58 | 0.55 | 0.6 | 0.43 |
| *ABCB1*  rs2032582  A>C | 0.67 | 0.62 | 0.57 | 0.61 | 0.98 | 1 | 0.57 | 0.54 | 0.55 | 0.59 | 0.61 | 0.62 | 0.54 | 0.47 | 0.36 |
| *ABCB1*  rs2032582  A>T | 0.01 | 0.05 | 0.02 | 0.02 | 0.001 | 0 | 0.06 | 0.05 | 0.05 | 0.12 | 0.03 | 0 | 0 | 0.13 | 0.05 |
| *SLC45A2*  rs35395  T>C | 0.54 | 0.39 | 0.96 | 0.87 | 0.24 | 0.2 | 0.5 | 0.66 | 0.42 | 0.18 | 0.67 | 0.68 | 0.5 | 0.12 | 0.22 |
| *XRCC1*  rs25487  T>C | 0.81 | 0.74 | 0.63 | 0.58 | 0.89 | 0.89 | 0.69 | 0.63 | 0.73 | 0.69 | 0.71 | 0.74 | 0.73 | 0.77 | 0.66 |
| *XRCC3*  rs861539  G>A | 0.01 | 0.22 | 0.39 | 0.4 | 0.19 | 0.19 | 0.23 | 0.32 | 0.17 | 0.06 | 0.32 | 0.31 | 0.19 | 0.07 | 0.21 |
| *ERCC2*  rs13181  T>G | 0.28 | 0.24 | 0.36 | 0.31 | 0.19 | 0.18 | 0.22 | 0.25 | 0.19 | 0.17 | 0.24 | 0.27 | 0.23 | 0.08 | 0.35 |
| *MGMT*  rs10764896  G>A | 0.50 | 0.6 | 0.51 | 0.49 | 0.63 | 0.73 | 0.49 | 0.48 | 0.46 | 0.45 | 0.54 | 0.48 | 0.47 | 0.78 | 0.56 |
| *MGMT*  rs11016885  T>C | 0.19 | 0.26 | 0.35 | 0.35 | 0.07 | 0.04 | 0.22 | 0.27 | 0.22 | 0.11 | 0.25 | 0.14 | 0.19 | 0.38 | 0.31 |
| World: N=2504, EUR: European N=503, IBS: Iberians N=107, AFR: African N=661, YRI: Yorubas N=108, AMR: American N=347, CLM: Colombians N=94, MXL: Mexicans N=64, PEL: Peruvians N=85, PUR: Puerto Ricans N=104, LAC1: Latin American individuals with Afro-Caribbean Ancestry N=variable sample size depending the SNV, LAC2: Latin American individuals with mostly European and Native American Ancestry N=variable sample size depending the SNV, SAS: South Asians N=489, EAS: East Asians N=504. Data collected from 1000 Genome Project. | | | | | | | | | | | | | | | |
